# Qualitative evidence synthesis for complex interventions and guideline development: clarification of the purpose, designs and relevant methods

**DOI:** 10.1136/bmjgh-2018-000882

**Published:** 2019-01-25

**Authors:** Kate Flemming, Andrew Booth, Ruth Garside, Özge Tunçalp, Jane Noyes

**Affiliations:** 1 Department of Health Sciences, Faculty of Science, The University of York, York, UK; 2 School of Health and Related Research, University of Sheffield, Sheffield, UK; 3 European Centre for Environment and Human Health, University of Exeter, Truro, UK; 4 Department of Reproductive Health and Research including UNDP/UNFPA/UNICEF/WHO/World Bank Special Programme of Research, Development and Research Training in Human Reproduction (HRP), World Health Organization, Geneva, Switzerland; 5 School of Social Sciences, Bangor University, Bangor, UK

**Keywords:** health services research, systematic review, qualitative study

## Abstract

This paper is one of a series exploring the implications of complexity for systematic reviews and guideline development, commissioned by the WHO. The paper specifically explores the role of qualitative evidence synthesis. Qualitative evidence synthesis is the broad term for the group of methods used to undertake systematic reviews of qualitative research evidence. As an approach, qualitative evidence synthesis is increasingly recognised as having a key role to play in addressing questions relating to intervention or system complexity, and guideline development processes. This is due to the unique role qualitative research can play in establishing the relative importance of outcomes, the acceptability, fidelity and reach of interventions, their feasibility in different settings and potential consequences on equity across populations. This paper outlines the purpose of qualitative evidence synthesis, provides detail of how qualitative evidence syntheses can help establish understanding and explanation of the complexity that can occur in relation to both interventions and systems, and how qualitative evidence syntheses can contribute to evidence to decision frameworks. It provides guidance for the choice of qualitative evidence synthesis methods in the context of guideline development for complex interventions, giving ‘real life’ examples of where this has occurred. Information to support decision-making around choice qualitative evidence synthesis methods in the context of guideline development is provided. Approaches for reporting qualitative evidence syntheses are discussed alongside mechanisms for assessing confidence in the findings of a review.

**Summary box**

- Qualitative evidence syntheses (QES) enable researchers to gain a greater understanding of individual’s experiences, views, beliefs and priorities for healthcare.
- QES are beginning to be incorporated in the development of guidelines for complex interventions.
- Detail is provided on how QES can help explain the complexity that can occur in relation to both interventions and systems.
- Guidance is given for the choice of QES methodology in the context of guideline development for complex interventions.
- Guideline producers are encouraged to consider the role of QES for reviews and guidelines on complex interventions implemented in complex systems.

## Background

This paper is one of a series exploring the implications of complexity for systematic reviews and guideline development, commissioned by the WHO. The paper specifically explores the role of qualitative evidence synthesis (QES), the types of questions likely to be addressed and the methods available in the context of guideline development for complex interventions and systems. It offers options for guideline developers to assist in making informed decisions about which QES methods to adopt.

Qualitative evidence synthesis (QES) is an umbrella term for the methodologies associated with the systematic review of qualitative research evidence, conducted either as a stand-alone review or as a part of a review of complex interventions, systems or of guideline development.^1^ The aim of a QES is to bring together the findings from qualitative research in order to establish a greater understanding of issues, often of a subtle or sensitive nature, that primary qualitative research frequently addresses. QES can provide rich interpretations relating to the impact of a condition, intervention or policy on the lived experiences and feelings of those involved.

Methods for conducting QES have developed against a backdrop of increasing demand from decision makers for evidence that goes beyond ‘what works’; a form of evidence traditionally established through systematic reviews of quantitative evidence, particularly reviews of randomised controlled trials (RCT). It is increasingly recognised that healthcare provision involves complex, multifactorial decisions which may require more than this original ‘rationalist’ model of synthesis can provide.^2^

In understanding why a QES can have a central role within guideline development for complex interventions or systems, it is important to appreciate the purpose of qualitative research, on which a QES is reliant. Qualitative research is generally interpretative in nature and through this seeks to develop understanding of and explanation for the behaviours, experiences and interactions of individuals and the social contexts in which these occur. Within the broad context of health, qualitative approaches can help determine the attitudes, beliefs and perspectives of patients, carers and clinicians to a condition, intervention, policy, context and structure of healthcare; can help understanding of the interpersonal nature of caregiver and patient relationships and behaviours; or enable insight into an illness experience.^3^ The data that are collected within qualitative research are predominantly text-based, narrative accounts commonly collected via interviews, focus groups or documentary analysis.

A QES, when used within clinical, public health, policy and health systems, can explore:

- Health-related behaviours or experiences of illness.
- Why and how a policy or intervention works.
- Appropriateness or acceptability of interventions.
- Barriers and facilitators to implementation of interventions.
- Gaps in primary qualitative research evidence, for example, gaps about knowledge of the acceptability of intervention.

## Future research priorities as a result of identifying gaps in evidence

One of the strengths of QES is the ability to bring together multiple perspectives, including contradictory viewpoints, which may not be represented within a single study alone.^1^ Additionally, the process of a QES enables researchers to ‘go beyond’ the findings of individual qualitative studies, by producing something that is greater than their simple sum.^1^ It is possible for a QES to produce a nuanced, in-depth and extremely useful picture of patient, carer and other health system users’ experiences, beliefs and priorities.^1^

In addition QES can, in relation to the specific focus to this paper, help inform the evaluation and implementation of complex interventions by:

- Exploring intervention complexity.
- Examining the context and success or failure of implementation.
- Determining the impact of wider health system issues.
- Exploring issues with fidelity, dose, reach, equity, process and outcomes.

Therefore, QES can be useful within a guideline development process by helping to explain the complexity that can occur in relation to interventions; whether this complexity exists within the intervention itself (when it is external to its target population), or within the system in which the intervention is to be implemented (understanding the dynamic properties of the context into which the intervention is to be introduced).^4^ A detailed explanation of a complex intervention perspective and how this differs from a complex systems perspective is provided elsewhere in this series.^4^ In considering the role of QES within the complex interventions/systems field it is important to remember that there is not, as yet, a distinct boundary between the two approaches within this rapidly evolving area.^4^ This paper explores how QES might contribute to the development of guidelines of complex interventions/systems and outlines different approaches that might be used to conduct such syntheses.

## Aspects of complexity addressed by QES within a guideline context

Depending on the purpose of the review, and drawing on the first paper in the series,^4^ there are a number of possible ways in which using a QES can help explain complexity within the context of guideline development (box 1).

Box 1 **Ways in which a qualitative evidence synthesis (QES) may help address elements of complexity**

- Develop a theory of why and how an intervention (complex or simple) works.
- Explore the experiences of recipients or providers of healthcare.
- Explore the experiences of living with a condition, which can impact on the feasibility and acceptability of an intervention.
- Examine the factors affecting implementation, including context.
- Determine how components of complex interventions work to produce effects.
- Establish how and why the implementation of interventions varies across contexts.
- Examine how a system changes when a complex intervention is introduced.
- What explains changes in the system over time.

An example of the way in which recipient experiences of a service can be integrated into the development of a guideline is demonstrated through the recent WHO recommendations on antenatal care for a positive pregnancy experience.^5^ A QES was undertaken as part of the guideline development process in order to identify outcomes of importance from the perspective of pregnant women, to inform the prioritisation of critical outcomes as well as the questions identified for the guideline.^5^

A QES can be conducted separately or can be integrated with some form of quantitative synthesis. Within a guideline development process, findings from a QES will often be integrated with evidence of effectiveness in an evidence to decision (EtD) framework, used to formulate recommendations.^6^ The WHO-INTEGRATE framework reported in this series^7^ incorporates six substantive criteria to ensure that all factors of relevance to a health decision are systematically considered (box 2), all of which have the potential to be informed by evidence from a QES. Sometimes it is relevant for a systematic review to combine qualitative and quantitative data; the underpinning philosophies and approaches for conducting mixed methods synthesis are detailed in another paper in this series.^8^

Box 2 **Criteria from the WHO-INTEGRATE evidence to decision framework that may be informed by qualitative evidence synthesis (QES)^7^**

- Balance of health benefits and harms.
- Human rights and sociocultural acceptability.
- Health equity, equality and non-discrimination.
- Societal implications.
- Financial and economic considerations.
- Feasibility and health system considerations.

## The importance of the research question in guidelines of complex interventions

Guidelines may be developed for many different purposes, including to inform clinical, public health and health system programmes and policies. Within the guideline process the research question underpins the purpose and, ultimately, the methods of a review. Unlike the typically fixed Population-Intervention-Comparison-Outcome question of quantitative synthesis, a QES question may be used as either the ‘compass’ or the ‘anchor’ for the review.^9^ The focus of the question for a QES within a guideline development process will depend on the potential nature of the complexity within the guideline (intervention or systems); examples of this are detailed in table 1. In some circumstances the review question may remain open to modification, with definitions of constructs occurring either late in the process or being a product of the review itself.^10^ Where the review is linked to, or is to be integrated with, a QES, it is more likely that the question will be ‘anchored’ from the outset.^9^ However, even where the quantitative and qualitative reviews are linked to the same guideline question, it may be helpful to extend the scope of searching for the QES beyond studies linked to specific interventions in order to fully explore the extent of complexity associated with the guideline.^11–13^

**Table 1:** Examples of the types of questions associated with complex interventions, systems and evidence to decision (EtD) frameworks

Aspect of complexity or complex interventions of interest to the guideline^4 7^	Possible questions that could be asked in a QES
What ‘is’ the system? How can it be described?	What are stakeholders’ interpretations of the current system?
Interactions between components of complex interventions	How do the components work in combination to produce effects?
Interactions of interventions with context	How and why does the implementation of an intervention vary across contexts?
System adaptivity: how does the system change?	Why does the system change in the way it does?
Emergent properties	What events were anticipated/unanticipated and what was the impact of these?
Multiple (health and non-health) outcomes	Why were outcomes not as anticipated?
Balance of health benefits and harms	To what extent do stakeholders value different outcomes?
Sociocultural acceptability of the intervention	What are stakeholders’ views about acceptability, or preferences or appropriateness of the intervention?
Feasibility and health system considerations	What aspects of the health system influence implementation of the intervention?

QES, qualitative evidence synthesis.

Further guidance on determining the focus of a QES to inform or explain effectiveness and complexity is provided elsewhere.^14^ The following steps outline how to identify the focus of a QES^14^:

1. Problem framing: Identifying the issues that need addressing.
  QES is used in reviews of complex interventions and guideline development to increase understanding of aspects such as context, implementation and views of a complex intervention. As a result, the issues that need addressing are directly linked to the need for evidence that describes or explains the phenomenon. There are multiple ways to frame a problem which subsequently inform the direction a review may take. A transparent process is recommended, as the review question may be revised on the basis of preliminary findings from the problem framing.^14^
  Involving stakeholders in problem framing is key, in order to explore ‘What’, ‘For whom’ and ‘Why’ and in ‘What context’ the problem exists. Through consultation with stakeholders, issues such as acceptability and implementation challenges will become apparent.
2. Constructing a preliminary framework or logic model to illustrate relationships.
  The use of theoretical frameworks or logic models in reviews of complex interventions/systems is becoming increasingly common across all types of QES. Such frameworks or models seek to present relationships between the problem, its explanatory evidence, how it may be implemented and its outcomes which, in turn, inform the focus and direction of the review.^8 15–17^ Theoretical frameworks are useful in explaining the possible relationships between concepts that are the focus of a review/guideline in general terms. More pragmatic illustrations of how the components of an intervention, programme or guideline may work together in a specific population and context are presented in logic models.^14^
3. Developing an understanding of context: Selecting a contextual frame that is appropriate to the complexity of the review question.
  One of the fundamental purposes of a QES conducted with a complexity perspective is to develop an understanding of relevant contextual factors. Knowledge of such factors will inform decisions on the ultimate scope of the search, inclusion and exclusion criteria and later considerations of transferability. How context may be addressed in relation to question formulation is explained in more detail in the paper by Booth *et al* in this series.^18^ Additionally, numerous contextual frames exist^14^ that can be used to help develop an understanding and impact of context in relation scope of the review; for example, the Context and Implementation of Complex Interventions framework is an overarching framework of interacting dimensions of context (including setting) and implementation.^19^
4. Identifying potential lines of inquiry: Considering which questions relate to effectiveness, implementation, feasibility, appropriateness of the intervention or strategy for implementation.
  The qualitative component of a review of complex interventions/systems may select one or more lines of inquiry in order to fully interrogate the question. Lines of inquiry addressed by the qualitative component can include questions about meaningfulness, appropriateness, feasibility, equity, affordability and implementation.
5. Searching to explore the knowledge base: Scoping the amount and type of knowledge and evidence currently available.
  Scoping the literature is vital to allow for the emergence of clear research questions to guide the reviewers.^20^ The scoping process seeks to determine the extent and availability of relevant research and also allows a preliminary assessment of its depth and quality to be made. Approaches to searching to establish the scope of a QES are well developed.^14 21^ After the initial scoping, further searches can be used to inform refinement of a review protocol.^14^
6. Formulating, focusing and refining questions: In relation to what is already known.
  Following scoping, the review question can be revisited or refocused where required prior to the main review searches being undertaken. Frameworks for structuring the question are well established,^9^ although their limitations are acknowledged with a new variant being proposed as part of this series.^18^ Within a guideline development process, the focus of the question for a review is commonly commissioned externally by the guideline developer. The use of an innovative question formulation framework can lead to qualitative evidence being used to contribute more nuanced insights within a complex interventions framework.
7. Developing the protocol: Using the preliminary questions and searching to understand how and why an intervention might work, taking into account the interactions between individuals and surrounding context.
  A protocol should be developed that presents an argument for the importance of the review, its relevance to the problems described and its potential utility to policymakers. The review question(s) should emerge naturally from the preliminary work and should include an approach to both the selection and appraisal of studies and synthesis that potentially enables reviewers to answer the questions.

## QES methods

The choice of method for a QES depends on the nature of the problem or research question being framed, the relationships determined within the framework, context and potential lines of enquiry and the literature available. Established and evolving methods for QES sit on a continuum between interpretative and integrative positions, the choice of which is determined by the focus of the review and the need to understand complexity within it.^22^

When considering the use of the methods for a QES it is helpful to recognise the ongoing variation in the use of terminology associated with QES; a number of terms have evolved as the approaches to QES have developed. QES is used as the overall term to refer to all methods that involve bringing together diverse types of qualitative evidence and is the term favoured by international groups such as the Cochrane Qualitative and Implementation Methods Group (CQIMG). This is due to its flexibility to incorporate broad types of qualitative evidence (eg, policy documents or grey literature reports), and also its acknowledgement that a separate set of methods, sensitive to the qualitative paradigm of research, is required.^9^ However, many alternatives exist, for example, the term ‘narrative synthesis’ has been adopted by both quantitative and qualitative approaches yet involves very different methods and outputs. Likewise, qualitative synthesis was historically used to describe synthesis of quantitative research, where a meta-analysis was not considered possible.

There is currently a range of methods available for QES, at varying levels of development and sophistication. Many draw on the principles of primary qualitative research and extend these to the level of synthesis. There are broad categories of QES on a continuum between integrative and interpretive approaches. Integrative synthesis focuses on summarising or aggregating data or themes, is a more deductive approach and often used when concepts or themes are clearly defined in primary research. Examples of such methods are thematic synthesis and framework synthesis. Interpretive synthesis allows for the development of concepts and theories grounded in the data contained in the primary studies; themes are developed inductively and the aim is to generate concepts and further develop theory, for example, using meta-ethnography. The approach to QES taken is dependent on the question and scope of the review.

### QES methods advocated for reviews of complex interventions

There are specific considerations when choosing a QES methodology within the context of reviews of complex interventions and guideline development. Another paper in this series outlines the philosophical approaches and methods for integration.^8^ A further consideration is that the qualitative findings need to be reported in a way that allows for use in an EtD framework for presentation to a guideline development group. In considering the choice of QES it is important to remember that the decision is multifactorial and cannot be driven by single criterion. Factors that underpin the choice of method include the understanding of complexity established through the development of the review focus through the seven steps outlined above. Additionally, CQIMG recommends that the choice of method should only finally be determined once the pool of evidence for the review is known and cautions against choice of method being prespecified.

With these caveats in mind, aspects to consider when choosing a QES methodology are presented below (table 2). These are adapted from the CQIMG guidance (CQIMG 2017).^23^ Each recommendation reflects the methodology’s fitness for purpose within the context of developing a guideline of complex interventions, not necessarily fitness for purpose to inform other forms of integrated or stand-alone reviews. The three methods identified are particularly suited to exploring aspects of complexity, but their ability to do this may be hampered by time, resources and the type of available data.^21^ Ultimately, the method chosen will depend on the level of conceptual and contextual detail in the studies. Higher levels of conceptual detail are described as ‘rich’, lower levels as ‘poor’. Higher levels of contextual detail are described as ‘thick’, lower levels as ‘thin’. The driver is the need to explore complexity with an appropriate method that can achieve the required type of synthesis and level of interpretation. Further worked scenarios illustrating how a review team might select an appropriate choice of method are found in a paper by Booth *et al*.^21^

Examples of how the methods have been used in the context of guideline development follow.

**Table 2:** Possible qualitative evidence synthesis methods to address aspects of complexity in systematic reviews and guidelines

Method	Explanation	Aspects of complexity that QES may be suitable to address^4 7^
Thematic synthesis (Thomas and Harden)^24^	*Pros:* Most accessible form of synthesis. Clear approach, can be used with data that are quite ‘thin’ to produce descriptive themes and where data are ‘thicker’ to develop descriptive themes into more in-depth analytic themes. These themes then need to be completely integrated within any quantitative synthesis. *Cons:* May be limited in interpretive ‘power’ and risks being used over simplistically and thus not truly informative for guideline development.	System adaptivity: Why does the system change Emergent properties Balance of health benefits and harms Sociocultural acceptability of an intervention
Framework synthesis (Oliver *et al*)^49^ Best fit framework synthesis (Carroll *et al*)^50^	*Pros:* Works well within reviews of complex interventions due to the extent of the complexity that any framework can accommodate, including representation of theory. The framework allows a clear mechanism for integration of qualitative and quantitative evidence in an aggregative way—see Noyes *et al*.^8^ Works well where there is broad agreement about the nature of interventions and their desired impacts. *Cons:* Requires work on how to identify, select and justify choice of framework. A framework may only be revealed as inappropriate once extraction/synthesis is under way. Risk of simplistically forcing data into a framework for expedience.	Interactions between components of complex interventions Interactions of interventions with context Multiple (health and non-health) outcomes Balance of health benefits and harms Sociocultural acceptability of an intervention Feasibility and health system considerations
Meta-ethnography (Noblit and Hare)^36^	*Pros:* Primarily interpretive synthesis method leading to creation of descriptive as well as new high-order constructs. Descriptive and theoretical findings can help inform guideline development. Requires primary studies to predominantly have data that are ‘thick’/rich. *Cons:* Complex methodology and synthesis process that requires a highly experienced team. Can take more time and resources than other methodologies. Theoretical findings may be a combination of empirical evidence, expert opinion and conjecture to form hypotheses. May not satisfy requirements for an audit trail (although new reporting guidelines will help overcome this).^45^ More work is needed to determine how CERQual^51^ can be applied to theoretical findings. May be unclear how higher level findings translate into actionable points.	What ‘is’ the system. How can it be described? Interactions between components of complex interventions System adaptivity: Why does the system change Balance of health benefits and harms Sociocultural acceptability of an intervention

CERQual, Confidence in the Evidence from Qualitative Reviews; QES, qualitative evidence synthesis.

#### Thematic synthesis

When a guideline tackles a question relating to sociocultural acceptability of a complex intervention, a QES using thematic synthesis may work well due to its ability to develop either descriptive or analytic themes to inform the guideline. Although ‘thematic synthesis’ is an umbrella term covering several different ways of synthesising qualitative evidence to develop themes, the various approaches follow similar methods and synthesis processes. Thomas and Harden’s version of thematic synthesis^24^ includes initially developing descriptive themes, which remain close to the primary studies. Where the quality and depth of data in the primary studies allows, this interpretation can be taken further to develop analytical themes which extend beyond the primary studies and generate new interpretive constructs, explanations or hypotheses. The integration of the qualitative and quantitative syntheses is used in another paper in the series on mixed methods reviews.^8^

#### Example of thematic synthesis used in a guideline process

The National Institute for Health and Care Excellence (NICE) commissioned a guideline on long-term rehabilitation after stroke.^25^ Stroke rehabilitation is a complex intervention consisting of components which address the physical, cognitive and psychosocial impacts of stroke, involving both the person who has experienced the stroke and their informal carers. It is delivered within a complex system of healthcare, drawing on a multidisciplinary team of healthcare professionals across both acute hospital and community-based settings. A central component of stroke rehabilitation is shared decision-making and goal setting between patients, their informal carers and health professionals. One aim of the guideline was to determine whether using goal setting with patients when planning their stroke rehabilitation activities leads to an improvement in psychological well-being, functioning and activity. This is an example of using QES to determine the ‘socio-cultural acceptability of an intervention’. NICE conducted a clinical evidence review which incorporated a synthesis of qualitative and quantitative evidence. The QES was conducted using thematic synthesis^1 25^ in which the themes from across the 17 included studies were aggregated.

The findings from the seven included quantitative studies highlighted that the standard approaches to goal setting for patients used by health professionals did not incorporate a patient-centred approach. The findings of the thematic synthesis identified that patients considered active participation in goal setting as vital to their rehabilitation.^26^ Incorporating the findings of the qualitative synthesis into the guideline led to its recommendations being driven by a patient-centred approach to stroke rehabilitation which ensured that people with stroke have meaningful and relevant goals for their rehabilitation which focus on activity and participation, are challenging but achievable in both the short term and long term, involving, where appropriate, the patient’s family or carer.

#### Framework synthesis and best fit framework synthesis

When a question in a guideline relates to feasibility or health system considerations, framework synthesis is a good choice of QES method due to the extent of the complexity the method can accommodate. As with thematic synthesis, the origins of framework synthesis can be traced to a corresponding method from primary research; in this case framework analysis. Framework analysis was developed by qualitative researchers in 1994 and is particularly suited for policy analysis.^27^ Framework synthesis shares several attributes with framework analysis being best suited to research with specific questions, a limited time frame and issues that have been identified a priori.^28^ Although generation of theory is a legitimate outcome from framework synthesis its main function is to interpret and integrate what is happening within a particular setting. Frameworks or best fit frameworks^29^ can be developed by or with stakeholders or derived from the published literature. There is a temptation for those using a framework to try to make the data fit, thereby reducing both the analytical value and its burden. However, when performed well, framework synthesis can result in considerable time savings, making it suited to a narrow policy window.^30 31^ Frameworks can derive from a pre-existing review, from a conceptual model, from a policy framework or from a logic model. So, for example, a framework from the WHO synthesis on barriers to facility-based delivery^32^ could be used to analyse studies derived from a single context (eg, Kenya). The chosen framework can also become a scaffold on which both quantitative and qualitative data can be juxtaposed, making it a suitable choice for reviews of complex interventions.

#### Example of framework synthesis used in a guideline process

Framework synthesis is currently the most commonly used approach in a guideline process and is a good choice when a question requires an understanding of complexity around feasibility and health system considerations. A QES of the barriers and facilitators to the implementation of lay health worker (LHW) programmes to improve access to maternal and child health^33^ is an example of a QES using framework synthesis to inform a guideline development. LHWs are individuals who provide a range of functions related to healthcare delivery. Commonly they are provided with training related to their role, although generally have no formal qualifications. The definition of ‘lay health worker’ is wide ranging and includes community health workers, village health workers, treatment supporters and birth attendants.^33^ It is both the wide-ranging nature of their role and the context in which they operate that makes LHWs a complex intervention.

This QES used a pre-existing framework (the Supporting the Use of Research Evidence framework^34^) to guide the synthesis as it provided a comprehensive list of possible factors that could inﬂuence intervention implementation. The synthesis was one of four syntheses of qualitative research that informed the WHO’s ‘Optimizing health worker roles to improve access to key maternal and newborn health interventions through task shifting’ guideline.^35^ The same framework was used across all four reviews making it possible to carry out an overarching analysis of factors inﬂuencing optimisation among different health worker groups.

When formulating the recommendations arising from the guideline, the findings from the QES were used to inform both acceptability considerations, that is, the likelihood that the delivery of the intervention would be acceptable to relevant stakeholders, and feasibility considerations such as: How feasible would it be to implement the intervention? What conditions would need to be in place? Which skills would be needed by the different types of health workers? Overall, rather than being seen as a health worker with less training, LHWs were commonly seen as a preferred type of health worker who bought different skills and were much valued by recipients.^33^

#### Meta-ethnography

Meta-ethnography is an explicitly interpretative approach to synthesis and aims to create new understandings and theories from a body of work.^36^ It is therefore particularly suitable for cases where there is a need to generate new explanations about a phenomenon and as such is highly relevant to developing guidelines of complex interventions when the question relates to identifying how or why the components of a complex intervention work together. It uses existing author interpretations of the data (sometimes called ‘second order constructs’, where the quotes from study participants are ‘first order constructs’—everyday ways of understanding the world) and looks for similarities and differences at this conceptual level. It uses translation, interpretation and comparison of study metaphors, constructs and ideas. It has been widely used in several areas of health research including exploring lay meanings of medicines taking,^37^ women’s experiences of menstrual bleeding^38^ and women’s experience of smoking during pregnancy.^39^ It draws on qualitative research paradigms that may be less easy to precisely describe and catalogue than some other approaches to QES.

#### Examples of meta-ethnographies contributing to a guideline process

Meta-ethnography is not yet commonly used in guideline development, particularly in relation to complex interventions and systems; more exemplars are required to demonstrate the value and application of meta-ethnography for this purpose. One example is that of Downe *et al*,^5^ who used a combination of framework synthesis and meta-ethnography in the recent WHO recommendations on antenatal care for a positive pregnancy experience. The QES was used to identify the processes and outcomes of antenatal care provision that are important to healthy pregnant women.^40^ The findings from this QES were influential in informing all the recommendations in the guideline.

A further example is of a meta-ethnography which explored how women’s circumstances and experiences influence their smoking behaviour in pregnancy, including their attempts to quit.^41^ The aspect of complexity of interest within this question is the ‘balance of health benefits and harms’. The meta-ethnography included 29 qualitative papers and represented the views of over 600 women who were predominantly from low socioeconomic backgrounds. The QES was used to inform the WHO recommendations on the prevention and management of tobacco use and secondhand smoke exposure in pregnancy.^42^ In particular, the QES provided detail of women’s values and preferences towards pharmacological and psychosocial interventions for smoking cessation in pregnancy. The use of this QES to inform guideline development is also an example of a pre-existing QES being used within the guideline development process. Where a QES already exists in relation to a guideline topic, it can be pragmatic to use it. Ultimately though, the findings that can be drawn from the QES will be determined by its closeness of fit to the guideline topic.

## Considerations for the use of findings from a QES in the context of guidelines for complex interventions

How the findings from a QES are used within the development of guidelines for complex interventions is dependent on the focus of the guideline and the nature of the complexity being considered. Outputs from QES vary, depending on the type of QES undertaken and this will be dictated by the nature of the guideline being developed. All findings can however be broadly placed on a continuum between ‘description’ and ‘interpretation’.^9^ A QES with predominantly descriptive findings, for example, from a thematic synthesis, or framework synthesis, generally incorporate less interpretation. Therefore, the output of such a QES can be as simple as a list of themes identified across the included studies which can be used by guideline developers to detail the needs, values, perceptions, behaviours and experiences of stakeholders within the guideline.

In a QES where the intention is to produce interpretative findings, such as that produced through a meta-ethnography, this requires that interpretations made by the reviewers are transparent, that is, it is clear how the interpretations arose and that they are plausible (ie, the findings are credible in light of the evidence provided).^9^ The output is commonly a contribution to a theory, further elaboration of a theory or a model that depicts the meaning of the data, which goes beyond the sum of the individual papers. Such higher order interpretations can be used to underpin guidelines in a theoretically informed way.

Transparent reporting has emerged as an increasingly important consideration for evidence synthesis; historically it has not been done well.^43^ Standardised reporting facilitates communication between different stakeholders, enables comparison of reviews and review proposals and is fundamental to the ultimate utility of the review. Clear reporting is particularly useful in guideline development but also for peer reviewers, funders and end users.^44^ Much work has been undertaken in recent years with regard to raising the standards of reporting of QES. Of the methods discussed in this paper, only meta-ethnography has its own reporting guideline, called eMERGe, which has a specific focus on the detailed synthesis process (http://www.stir.ac.uk/emerge/).^45^ Framework synthesis and thematic synthesis are accommodated by a generic reporting standard, the ‘Enhancing Transparency in the Reporting of Syntheses of Qualitative Research’ tool.^46^ This was the first reporting tool for QES and consists of 21 items within the five overarching categories. As a generic tool it documents the most frequently used methods for QES to which it might apply, acknowledging that the approaches and methodology for synthesis are usually driven by the research question posed.^46^

When undertaking a QES it is important to be able to assess the degree of confidence in which the key findings are a reasonable representation of the phenomenon of interest. The Grades of Recommendation, Assessment, Development and Evaluation-Confidence in the Evidence from Qualitative Reviews (GRADE-CERQual) approach^47^ has been developed to guide reviewers to assess confidence in findings from QES. The GRADE-CERQual approach has particular strength in overcoming some of the limitations of many quality appraisal tools in as much as there is a focus on the quality of analysis and data rather than on technical reporting issues.

GRADE-CERQual provides a transparent and systematic framework containing four components for assessing how much confidence to place in key findings: (1) the methodological limitations of the individual qualitative studies contributing to a review finding; (2) the coherence of the review finding; (3) the adequacy of data supporting a review finding; and (4) the relevance to the review question of the individual studies contributing to a review finding. A fifth component, dissemination (or publication) bias, may also be important and is being explored.

The use of GRADE-CERQual is particularly helpful when undertaking QES for guideline development of complex interventions/systems. As its approach is congruent with other GRADE approaches it can be easily integrated into decision-making processes. Its use can enable QES to address a range of issues of complexity, a process vital to integrating qualitative evidence within EtD frameworks, including establishing which outcomes are important to stakeholders; the acceptability and feasibility of interventions along with any unintended consequences of interventions; and key factors to consider regarding implementation.^48^

## Conclusion

QES has a key role to play in the development of guidelines for complex interventions. This paper provides a summary of what QES is, how it can contribute to systematic reviews and guidelines of complex interventions, and provides a series of options for QES methods to support guideline developers in making informed decisions depending on the type of complexity-related question being asked.
